# Novel optical coherence tomography findings in a patient with methanol toxicity: a case report

**DOI:** 10.5935/0004-2749.2022-0068

**Published:** 2024-03-05

**Authors:** Burcu Işık, Mehmet Giray Ersoz, Mehmet Dokur, Ismail Yıldız, Mehmet Sami Islamoğlul

**Affiliations:** 1 Istanbul Biruni University Hospital, Istanbul, Turkey

**Keywords:** Methanol, Toxicity, Tomography, optical coherence, Subretinal fluid, Epiretinal membrane

## Abstract

We report a case of acute methanol toxicity with unique optical coherence tomography findings. A 56-year-old man was referred to our ophthalmology clinic with a history of handmade vodka consumption and vision loss. On ophthalmologic examination, his vision was 20/100 in his right eye and 20/200 in his left eye. Bilateral mild optic disk hyperemia was detected on fundus examination. Because of the severity of systemic symptoms in such cases, it is very difficult to include optical coherence tomography in the ophthalmologic examination. However, we managed to perform optical coherence tomography and recorded shallow subretinal fluid and a prominent middle limiting membrane sign as acute retinal structural changes in the patient. The patient was treated with hemodialysis, intravenous ethanol, and sodium bicarbonate. On the fourth day of treatment, visual acuity improved to 20/20 in both eyes. In addition, the prominent middle limiting membrane sign and subretinal fluid disappeared. In this unusual case, retinal pigment epithelium damage and retinal ischemia may have contributed to the prominent middle limiting membrane and subretinal fluid, which are novel optical coherence tomography findings of methanol toxicity.

## INTRODUCTION

Methanol is a nonconsumable form of alcohol and a component of many chemicals, such as antifreeze, solvent, dye, perfume, and cologne. Methanol poisoning is an increasing public health problem in developing countries, and the most common form occurs via adulterated alcohol^(1)^. Alcohol and aldehyde dehydrogenase enzymes convert methanol to formic acid. Formic acid acts as the main cause of methanol toxicity by inhibiting cytochrome oxidase activity, which causes tissue hypoxia and mitochondrial damage^(2)^. Hypoxia has a severe cytotoxic effect on retinal ganglion cells and optic nerves. Thus, both retina and optic disk destruction may be observed in methanol toxicity^(3)^. Methanol toxicity leads to visual dysfunction, which can vary from a mild decrease in vision to total blindness. Other visual symptoms include scotomata, dyschromatopsia, and photophobia^(4)^.

Herein, we report the first case of a patient with acute methanol toxicity with subretinal fluid and a prominent middle limiting membrane (p-MLM) sign on optical coherence tomography (OCT).

## CASE REPORT

**Figure 1:**
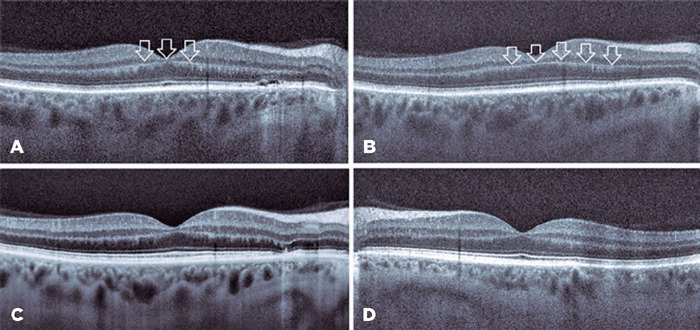
Optical coherence tomography (OCT) images. (A, B) Right eye before treatment. (A) Shallow subretinal fuid was seen at the nasal site, and the prominent middle limiting membrane sign (white arrows) appeared at the border of the outer plexiform layer and inner nuclear layer. (B) The prominent middle limiting membrane sign (white arrows) became more evident on this OCT scan. (C) Right eye after treatment. Foveal OCT scan showed that subretinal fuid and the prominent middle limiting membrane sign have disappeared. (D) Left eye after treatment. The foveal OCT scan was normal.

**Figure 2:**
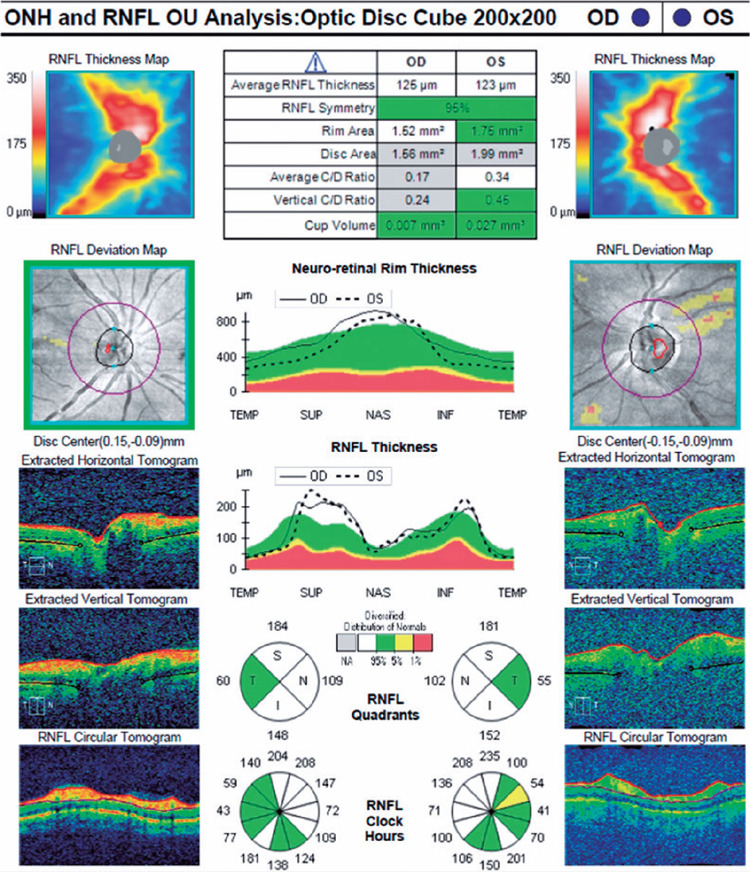
Bilateral retinal nerve fber layer analysis during the frst admission showed a signifcant increase in retinal nerve fber layer thicknesses.

A 56-year-old man was brought to the emergency department via ambulance with symptoms of labored breathing and blurred vision. His detailed history revealed homemade vodka consumption in the evening of the previous day. He was conscious and cooperative but had difficulty breathing (acidotic breathing). After his general examination, was administered at the initial loading dose (8 mL/kg/h) and 10 mL molar sodium bicarbonate (8.4%) as an I.V. push^(5)^. Afterward, he was referred to the ophthalmology department. Ophthalmologic examination revealed a best-corrected Snellen visual acuity (BCVA) of 20/100 in his right eye and 20/200 in his left eye. Pupillary direct and indirect light reflexes were strongly positive in both eyes. No relative afferent pupillary defect was present. Color vision was determined to be intact using Hardy-Rand-Rittler pseudoisochromatic plates. Anterior segment examination was normal in both eyes. Fundus examination demonstrated mild optic disk hyperemia in both eyes. OCT, retinal nerve fiber layer (RNFL) analysis, and fundus photography were performed. Due to the patient’s noncompliance, a visual field examination was not possible during the first ophthalmologic examination. Foveal OCT scans of the right eye indicated a p-MLM sign and shallow subretinal fluid between the fovea and optic disk. OCT was not clear in the left eye because the patient could not remain in position due to mild dyspnea (Figure 1). The RNFL thickness was increased significantly in both eyes (Figure 2).

Arterial blood gas analysis revealed that the patient had metabolic acidosis with a serum pH of 7.17 (reference range [RR], 7.35–7.45) and bicarbonate level of 11 mmol/L (RR, 22–29 mmol/L). He was admitted to the intensive care unit (ICU) and treated as per the guidelines^(5)^ with continuous hemodialysis, ethanol infusion titrated to a serum concentration of 1.0–1.5 mg/mL, I.V. bicarbonate, and I.V. folinic acid 50 mg every 6 h. Serum toxicology confirmed methanol toxicity with a methanol level of 42.9 mg/dL (RR, 0–0.05 mg/dL). The patient was discharged from the ICU with a serum pH level of 7.4 on day 4 and was referred to the ophthalmology department. OCT, RNFL, and visual field analysis were performed. Optic disk hyperemia disappeared on fundus examination. Although the RNFL thicknesses were the same in both eyes, the foveal OCT scans showed the complete disappearance of subretinal fluid and p-MLM in the right eye.

**Figure 3:**
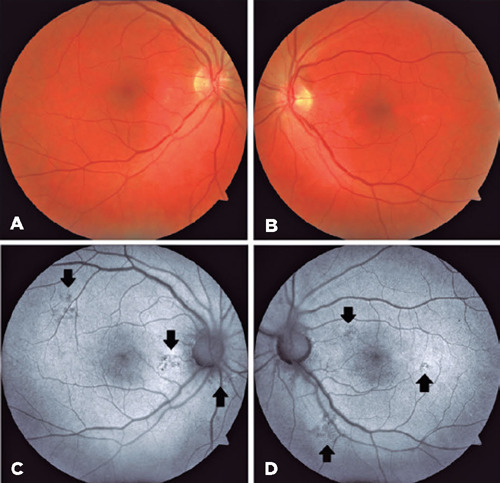
(A, B) Color photography of patient. Fundus autofuorescence images of the right (C) and left (D) eye after treatment. (C, D) Some hypoautofuorescence lesions (black arrows) indicating retina pigment epithelium damage were seen.

There were no pathologic OCT findings in the left eye. Fundus autofluorescence was performed, and multifocal extrafoveal hypoautofluorescence lesions were detected (Figure 3). Visual acuity recovered dramatically, and both BCVAs were 20/20. There were also no visual field defects.

## DISCUSSION

Both metabolic acidosis and formic acid result in methanol poisoning symptoms, and early treatment is critical. Metabolic acidosis mainly contributes to systemic toxicity, and ocular effects are thought to be a direct effect of formic acid. Ocular symptoms appear 6-48 h after consumption^(6,7)^. The main presentation of methanol toxicity is toxic optic neuropathy with or without optic disk and retinal swelling^(7)^. Both optic disks were hyperemic in our patient, and the increase in RNFL thickness was considered to be a result of methanolinduced optic neuritis.

Based on findings of electrophysiological tests and clinical features, methanol toxicity affects Müller cells, retina pigment epithelium (RPE), and photoreceptors^(8)^. Prior studies on methanol toxicity revealed OCT findings such as retinal thinning, inner nuclear layer microcysts in the chronic phase, swelling of the peripapillary nerve fiber layer, and accumulation of intraretinal fluid in the acute phase^(4)^.

We observed accumulation of subretinal fluid in our patient. Although the mechanism of subretinal fluid accumulation was not clear in this case, subretinal fluid might be followed by severe and acute dysfunction of retinal cells, such as photoreceptors and RPE. Ranjan et al. showed multifocal extrafoveal serous RPE detachments using fundus fluorescein angiography^(7)^. In our patient, fundus autofluorescence images revealed extrafoveal multifocal hypoautofluorescent lesions indicating RPE damage. His serum level was markedly high at 42.9 mg/dL, which made us suspect chronic methanol consumption. Extrafoveal serous RPE detachments are mostly asymptomatic. Accordingly, we assume that the recurrent extrafoveal RPE detachments in our patient resulted from these extrafoveal multifocal hypoautofluorescence lesions in the chronic phase of methanol exposure.

Foveal OCT scans also revealed the p-MLM sign, which is a hyperreflective line in the inner part of the outer plexiform layer resulting from acute retinal ischemia. It is thought that cytoplasmic swelling of predominantly bipolar cells sparing the rod and cone pedicles in the inner synaptic portion of the outer plexiform layer results in a hyperreflective line^(9)^.

According to some studies, corticosteroids improve the visual function of patients with methanol toxicity; however, the validity of this claim remains questionable^(6,10)^. We did not include corticosteroids in the treatment of this patient. Nevertheless, the subretinal fluid and p-MLM signs resolved after treatment. BCVA fully recovered to 20/20 in both eyes. We assume that the recovery of BCVA was due to the resolution of the retinal findings.

To the best of our knowledge, this is the first case of p-MLM and subretinal fluid accumulation detected in a patient with acute methanol toxicity.

In conclusion, this case revealed subretinal fluid and a p-MLM sign in a patient with acute methanol toxicity. We consider these novel OCT findings to be the result of RPE damage and retinal ischemia. Detecting and monitoring retinal structural changes related to methanol toxicity that were previously unknown is crucial for understanding and managing this devastating disease.
